# The Prognostic Value of Plasma Cell-Free DNA Concentration in the Prostate Cancer: A Systematic Review and Meta-Analysis

**DOI:** 10.3389/fonc.2021.599602

**Published:** 2021-03-11

**Authors:** Hongtao Liu, Yuzhen Gao, Somayeh Vafaei, Xiao Gu, Xiaoli Zhong

**Affiliations:** ^1^ Department of Graduate School, Dalian Medical University, Dalian City, China; ^2^ Department of Urology, Northern Jiangsu Hospital, Yangzhou University Clinical College, Yangzhou, China; ^3^ Department of Molecular Diagnosis, Northern Jiangsu Hospital, Yangzhou University Clinical College, Yangzhou, China; ^4^ Department of Molecular Medicine, Faculty of Advanced Technologies in Medicine, Iran University of Medical Sciences, Tehran, Iran; ^5^ Clinical Medical College, Yangzhou University, Yangzhou City, China

**Keywords:** prostate cancer, cell-free DNA, meta-analysis, diagnosis, prognosis

## Abstract

**Objective:**

By virtue of largely disparate clinical outcomes of prostate cancer (PCA), there is a pressing need to search for useful biomarkers for PCA prognosis. Cell-free DNA (cfDNA) is a promising biomarker for detecting, monitoring, and predicting survival of prostate cancer (PCA). However, the utility of total cfDNA quantitation in PCA in clinical setting remains elusive. Here, we performed a thorough meta-analysis to assess the prognostic value of cfDNA concentration for patients with PCA. In addition, we tested the possibility of the combination of PSA and cfDNA test results to improve the prediction power in PCA prognosis.

**Method and Materials:**

More than six databases, including PubMed, Web of Science, Medline, PMC, EMBASE and the Cochrane Library were searched. Results yielded all eligible articles from the date of inception to June 30, 2020. Continuous, diagnostic, and prognostic variables in cfDNA in PCA were included in the meta-analysis by STATA.

**Results:**

A total of 23 articles were enrolled in our meta-analysis: 69.6% (16/23) were related to diagnosis, and 56.5% (13/23) were related to prognosis. The pooled concentration of cfDNA in PCA patients was significantly higher than in the control group (SMD = 0.89, 95%CI = 0.53, 1.26), mirroring results for the prostate-specific antigen (PSA). For the detection test variables, the SROC with 95%CI was 0.87 (0.84–0.90) for cfDNA concentration. In terms of prognostic variables, the concentrations of cfDNA were significantly related with progression-free survival (PFS, logHR = 0.84 (95%CI0.39, 1.28) and overall survival [OS, log HR = 0.60 (95%CI0.29, 0.90)]. Lastly, the test showed no significant publication bias in the present meta-analysis, excluding the diagnostic meta-analysis.

**Conclusions:**

The concentration of cell-free DNA is high in the prostate cancer patients. The present study substantiates the prognostic value of the cfDNA concentration. High concentration cfDNA correlates with poor disease outcome of CRPC. The study cohort with large sample size is needed to evaluate the prognosis value of cfDNA in the future. We also emphasized that combination of PSA and cf DNA quantitation is important in future large individual meta study.

## Introduction

Prostate cancer (PCA) is the second most common malignancy in males worldwide and, it is the fifth leading cause of death in men globally. The progression of prostate cancer embodies a heterogenous entity (1). While the 5-year survival rate for the majority of patients with local prostate cancer is nearly 100%, the median survival rate of patients diagnosed with metastatic castration-resistant prostate cancer (mCRPC) is about 2 years (2). What is more, the clinical courses of mCRPC patients vary substantially (3). Over decades, PSA remains the predominant biomarker with the diagnostic and prognostic value in prostate cancer. Traditionally biomedical recurrence (the rise of PSA after the treatment), in addition to the tumor stage, pathological Gleason score was considered to provide useful prognostic information. However, recently there is a loss of discriminatory power for these clinical factors in PCA prognosis due to a wide adoption of PSA screening in many countries (4). The PSA screening has led to early identification, overdiagnosis, and overtreatment of the prostate cancer (5–7). There is an urgent need for new biomarkers to distinguish the high risk for treatment failure and death from indolent prostate cancers, especially at the time of diagnosis.

The AR gene is frequently altered in castration resistant metastatic prostate cancer. The mutations and copy number variations of AR gene have shown the link with both of the primary and the acquired resistance to the systemic therapy that targets the androgen–androgen receptor axis (8). The splice variant AR-V7 is also associated with the resistance to hormonal therapy. Somatic alteration of AR gene detected from the plasma cell free DNA in PCA patients might be predictive of resistance to therapy for metastatic prostate cancer patients. Hence, incorporation of cfDNA tests into the clinics has gained importance in a new era of precision prostate cancer therapy. Circulating cell-free DNA (cfDNA) is the diminutive DNA fragment in blood stream, which was mainly excreted by tumor. Therefore, it might be an approximate reflection of tumor burden. The concentration of cfDNA in the blood circulation can be comparably easy to measure in the clinical laboratories. The cfDNA quantitation might have the potential to become a new biomarker replacing or compensating PSA tests in prognostic evaluation of PCA (9). Even though most of previous studies focused on the discovery of genetic and epigenetic alterations of cell free DNA in the PCA disease, utility of total cfDNA level thus far remains controversial in clinical settings (10). Herein we consequently present a systematic review and a meta-analysis in regard to the utility of cfDNA, specifically focused on cfDNA concentration, for the prognosis evaluation of prostate cancer. In order to provide an insight of the biomarker power of serological cfDNA level in PCA disease, we parallelly analyzed the PSA values, which were assessable from the same literatures assembled for the meta-analysis.

Currently, cell-free DNA assay is extracted from a small volume of serum or plasma material (11). A great deal of previous researches on cfDNA have focused on the quantitative aspects of circulated cell-free DNA (12), methylation of the corresponding gene, its copy number variations (13, 14), and the serum DNA sequence. A number of reports have suggested that the cfDNA quantification test could represent a promising candidate biomarker for the early diagnosis of prostate cancer and the monitoring of cancer recurrence. Although a number of studies have contributed to our understanding of cfDNA levels, there remains considerable difficulty when attempting to compare such studies, particularly in regard to the DNA levels reported. Therefore, the present meta-analysis seeks to obtain data on the concentration of serological cfDNA in patients with PCA, which will help to address these research gaps.

## Materials and Methods

### Data Sources and Search

This literature search was guided by the recently published PRISMA statement (15). For the meta-analysis, the authors searched PubMed, Web of Science, Medline, PMC, EMBASE, and the Cochrane Library to retrieve all eligible articles from the date of database inception to June 30, 2020. The search heading terms and keywords included “prostate cancer”, “cfDNA”, “diagnosis” and “prognosis”. Additional articles were identified by manually reviewing the references of included articles. No languages restrictions were applied. Other details could be seen in **Table S1**. When judged necessary, the authors of the included articles were contacted.

### Inclusion and Exclusion Criteria

This meta-analysis focused on exploring the effect of diagnostic and prognostic values in patients with PCA. Therefore, we assessed the clinical values of cfDNA for PCA patients through three designs, which were difference, diagnosis, and prognosis with PFS and OS. These studies were included if they met the following criteria: (a) studies that evaluated indicators originating from circulating cfDNA should be detected in plasma or serum; (b) quantitative and qualitative data were presented on prostate cancer or control groups for assessing the personal difference and describing or calculating sensitivity and specificity values; (c) studies provided enough information to obtain HRs directly or indirectly for overall survival or progression-free survival. Studies meeting any of the following criteria were excluded: (a) the articles were not containing cfDNA data for the control group, if the design of an article was defined as diagnosis; (b) reviews, letters, technical reports, case reports, comments; (c) studies consisting of less than five prostate cancer patients.

### Data Extraction

Two investigators (Liu and Gao) independently assessed the titles and abstracts of retrieved citations to identify potentially relevant studies, reviewed the main reports and supplementary materials. Any disagreements were solved by consensus. Related data was extracted into a table including the following information: first author, publication year, country and region, evaluating indicator, patient’s characteristics (age, the number of patients, clinical stage, and the status of bone or LN metastatic, *e.g.*) and detection methods of cfDNA. Additionally, the hazard ratio for overall survival and progression-free survival were also presented in the other form. The articles with attachments were also downloaded, including the original data. Other details on the diagnostic indicators and prognostic are represented in **Table 1**.

**Table 1:** Baseline characteristics of the eligible 23 articles.

Cations	Authors	Year	Country	Patients type0 (Cancer/Control)	Cancers Treatments	Number (cancer/control)	TP/FN/FP/TN	Detection methods	Meta-Type
(16)	Boddy et al.	2005	UK	PCA/BPH+Health	NA	78/25	NA	QPCR	Dia
(17)	ALLEN et al.	2004	England	PCA/BPH	NA	15/10	13/2/2/8	QPCR	Dia
(18)	Altimari et al.	2008	Italy	PCA/Health	NA	64/45	51/13/8/37	QPCR	Dia
(19)	Belic et al.	2018	Austria	mCRPC/NA	Abiraterone or enzalutamide	14/NA	NA	QPCR	PFS
(20)	Cherepanova et al.	2008	Russia	PCA/Health	Untreated patients	5/22	NA	PicoGreen	Dia
(21)	Chuns et al.	2006	Canada	PCA/BPH	NA	142/19	NA	NanoDrop	Dia
(22)	Ellinger et al.	2008	Germany	PCA/BPH+Health	Radical prostatectomy	168/53	59/109/19/34	QPCR	Dia
(23)	Goodall et al.	2017	UK	PCA/NA	The PARP inhibitor olaparib	42/NA	NA	PicoGreen Quantification	OS,PFS
(24)	Gordian et al.	2010	USA	PCA/BPH	NA	89/104	40/49/32/72	QPCR	Dia
(14)	Hendriks et al.	2018	USA	CRPC/NA	NA	47/NA	NA	Qubit	OS
(25)	Reis et al.	2015	USA	PCA/BPH	NA	34/48	17/17/5/43	QPCR	Dia
(26)	Jung et al.	2004	Germany	PCA/NA	Hormonal therapy + radical prostatectomy or radiotherapy	91/93	23/68/12/81	QPCR	OS
(27)	Kienel et al.	2015	Germany	CRPC/NA	taxan-based chemotherapy	59/NA	NA	NanoDrop	OS,PFS
(28)	Wroclawski et al.	2013	Brazil	PCA/BN	Untreated patients	133/33	88/45/4/29	PicoGreen Quantification	Dia, PFS
(29)	Mehra et al.	2007	Netherlands	CRPC/BPH	NA	75/14	NA	RT-PCR	Dia, OS, PFS
(30)	Papadopoulou,et al.	2006	Greece	PCA/Health	chemotherapy	12/13	7/5/1/12	RT-PCR	Dia
(31)	Ponti.et al.#1	2018	Italy	PCA/Health	NA	18/13	18/0/0/13	Qubit	Dia
(32)	Schutz et al.	2015	Germany	PCA/Health	NA	204/207	NA	Sequencing	Dia
(33)	Schwarzenbach et al.	2009	Greece	PCA/Health	NA	81/10	NA	NanoDrop	Dia
(34)	Torquato et al.	2019	USA	mCRPC/NA	enzalutamide and abiraterone	62/NA	NA	NGS	OS, PFS
(35)	Vandekerkhov et al.	2018	USA	mCSPC	ADT	53/NA	NA	sequence	Dia, OS
(36)	Wyatt et al.	2016	UK	CRPC/NA	Enzalutamide	65/NA	NA	Sequencing	Dia, PFS
(37)	Annala et al.	2018	Canada	mCRPC/NA	enzalutamide and abiraterone	202/NA	NA	sequence	OS, PFS

NA, not available; cfDNA, cell free DNA; TP, true positive; TN, true negative; FP, false positive; FN, false negative; QPCR, Real-time Quantitative PCR Detecting System; Pico Green, Quant-iT PicoGreen; BN, Biopsy-negative; PCA, prostate cancer; BPH, benign prostatic hyperplasia; mCPRC, Metastatic Castration-Resistant Prostate Cancer; mCSPC, Metastatic Castration Sensitive Prostate Cancer; Dia, diagnostic design; OS, overall survival; PFS, progression free survival; PSA, prostate specific antigen; ADT, Androgen Deprivation Therapy.

### Quality Assessment

For all studies, we used the quality of included related studies basing on the Newcastle–Ottawa scale criterion, where five to nine stars means high quality and one to four stars means low quality. Additionally, according to the types of the included articles, they were divided into two scales, “case–control” study NOS Scale and “cohort” study NOS Scale in **Table S2**.

### Statistical Analysis

To better present the clinical values of cfDNA in prostate cancer, we used a variety of statistical methods step-by-step. Firstly, the difference between prostate cancer and the control group was assessed by the standard mean difference (SMD) for the articles reporting the value of cfDNA. P <0.05 was considered as a significant difference in the value between the two groups. Secondly, if possible, according to the original data of special researches, the correlation between PSA and cfDNA was also calculated. Then, the available data were translated into diagnostic numbers (TP, FP, FN, TN) in two groups of diagnostic studies. These numbers were used to calculate pooled sensitivity, specificity, positive likelihood ratio (PLR), negative likelihood ratio (NLR), diagnostic odds ratio (DOR), area under the curve (AUC), and corresponding 95% confidence intervals (95% CI). Summary ROC curves (SROCs) and AUCs of the SROC (AUSROC) were also measured. The threshold effect was detected in order to find the difference of that in different studies. For the prognostic value of cfDNA, the HR with 95%CI values for the progression-free survival and overall survival in prostate cancer were pooled in the prognostic studies. In the next step, the I² statistic was used to investigate the heterogeneity among these studies. I² >75% indicated a large inconsistency; the random-effect model was used to pool the data; Otherwise, I^2^ <50% indicated a small inconsistency; a fixed-effect model was selected. Regression analyses were also conducted in this step according to clinical variables. Lastly, the Begg’s test was used to assess the publication bias of pooled results of more than ten articles which focused on the difference and prognosis, and the Deeks-funnel plot was used to test the diagnostic results of all the articles. P <0.05 was considered as a statistical significance, and all the statistics were performed using STATA (version 14, USA) and R (Vienna, Austria, version 3.4.6).

## Results

### Search Results and Study Assessment

**Figure 1:**
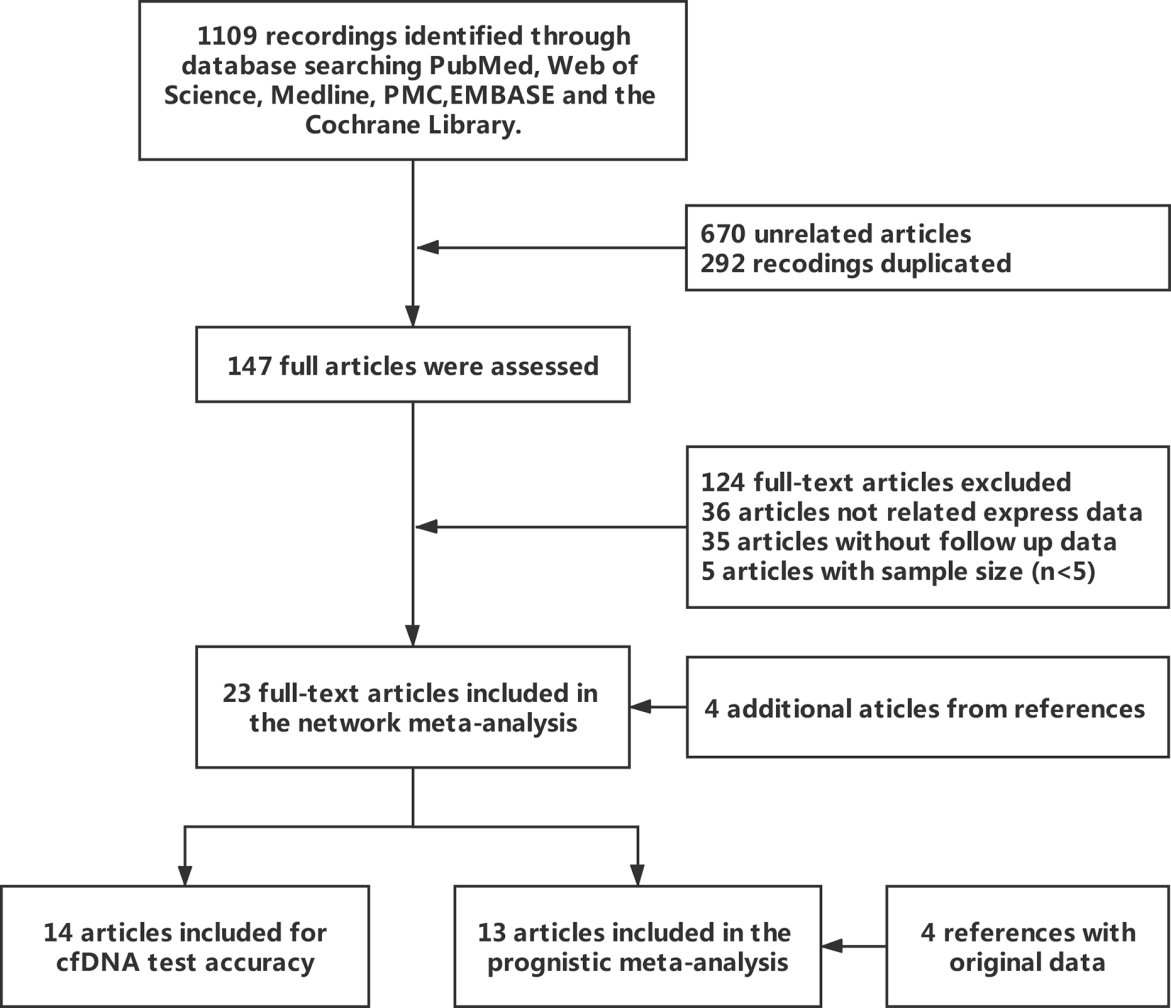
Flowchart of study inclusion and exclusion for meta-analysis.

The explicit search strategies of this meta-analysis were also presented in the **Table S1**, and the flowchart of the search was shown in **Figure 1**. Of 1,109 relevant publications which were identified on the database searches, 147 potentially eligible articles were retrieved for full-text review. A further 124 publications were excluded because of lack of the reported outcome (n = 36), absence of follow-up data (n = 35), low number of samples (n = 5). Additionally, four articles were added by manual in the references. Lastly, the remaining 23 articles were enrolled into two main types of meta-analysis; 69.6% (16/23) were for the difference or diagnosis meta-analysis between patient group and control group, 47.8% (11/23) were for the prognostic meta-analysis with categorical variables.

All articles were published from 2000 to 2020, and almost the articles are from Europe and America. The concentration was detected to explore the diagnostic values of that in the cancer group (n =1,236) and control group (n = 616). For the prognosis, 843 and 140 patients were respectively included in the meta-analysis. The other detailed characteristics of the 23 included studies are summarized in **Table 1**.

For quality accession, 10/23 articles, as a case–control cohort study, were assessed six to nine by the Newcastle–Ottawa scale criterion with the average seven scores (**Table S2**). Another 13/23 articles, in which 23.1% (3/13) articles were six points, and the remaining 76.9 (10/13) were seven were evaluated additionally. In a word, the articles, which were included in our research, were of high quality.

### Diagnostic Efficiency of the Concentration of cfDNA Assay Between the PCa and Control Group

**Figure 2:**
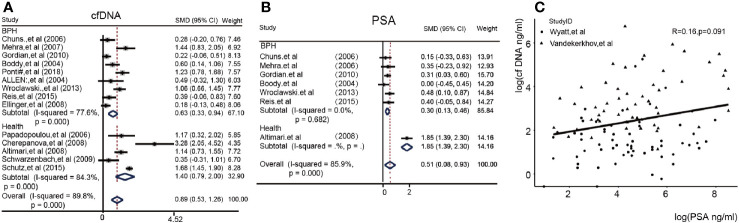
The heterogeneity of cfDNA and PSA values between the PCA and control group (cfDNA, cell free DNA; PSA, prostate-specific antigen.) **(A)**: Forest plots of the concentration of cfDNA; **(B)**: Forest plots of PSA; **(C)**: Correlation between cfDNA and PSA in Alexander’s and Vandekerkhov’s article.

We at first sought to identify the detection power of the cfDNA test. **Figure 2A** showed the difference between the PCA group and the Control group in 14/23 article. We found that the Subtotal SMD [0.89 (95%CI = 0.53, 1.26)] with a high I^2^ (89.8%) for the concentration of cfDNA in the PCA patients was significantly higher than the control group (BPH or Health) in a total of 14 articles. Subgroup analysis could also be seen in this. Obviously, healthy people as control had a higher difference [SMD 1.40(0.79, 2.00) *vs* 0.63(0.33, 0.94)] than BPH patients as control. In addition, we compared the PSA value in enrolled seven articles and found that the overall SMD was 0.51(95%CI = 0.08, 0.93) in **Figure 2B**. **Figure 2C** showed log(cfDNA) was not related with log(PSA) in two articles with detail data (R = 0.16, p = 0.091). Then, we further analyzed the diagnostic value of cfDNA. Fortunately, the cfDNA had a median pooled sensitivity (0.69,95%CI 0.46–0.85) and a high specificity (0.86,95%CI 0.76–0.92) (**Figures S3A, S3B**). Eventually, the SROC with 95%CI of cfDNA’s concentration were 0.87 (0.84–0.90) in the **Figure S3C**. Further analysis, we also calculated the pooled positive likelihood ratio (PLR), negative likelihood ratio (NLR) and diagnostic odds ratio (DOR) in the **Supplemental Figure S4**.

### Prognostic Value of cfDNA Concentration in Patients With PCA

**Figure 3:**
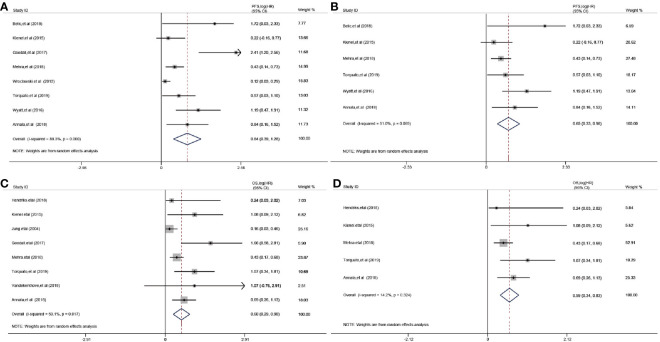
Prognostic value of cfDNA in prostate cancer. **(A)**: Forest plots for PFS; **(B)**: Forest plots for PFS in CRPC (Castration-Resistant Prostate Cancer); **(C)**: Forest plots for OS; **(D)**: Forest plots for OS in CRPC.

**Figure 4:**
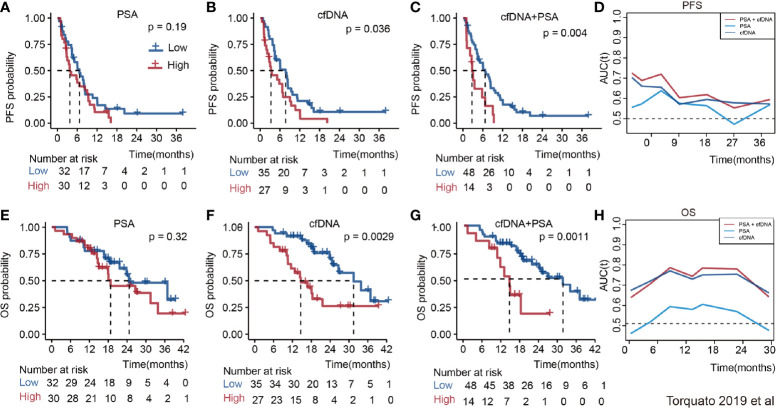
The prognosis value of PSA, cfDNA and the combination of them in the Torquato’s cohort. **(A)**: PSA for PFS; **(B)**: CfDNA for PFS; **(C)**: PSA + cfDNA for PFS; **(D)**: Time-dependent ROC of them for predicting PFS; **(E)**: PSA for OS; **(F)**: CfDNA for OS; **(G)**: PSA + cfDNA for OS; **(H)**: Time-dependent ROC of them for predicting OS.

The relationship between cfDNA and biochemical recurrence free survival was invested by meta-analysis. Cautiously, result showed that high concentration of cfDNA correlated with the PSA recurrence (HR 1.23 95%CI(1, 1.45), **Figure S5**) in PCa patients of three articles. Then, we tested the prognostic value for the concentration of cfDNA. The pooled analysis showed that cfDNA was associated with poorer PFS (log(HR) = 0.84, 95%CI[0.39, 1.28]; **Figure 3A**), with a statistical significance in between-study heterogeneity (I^2^ = 88.3%, P < 0.001). For OS, similarly, high concentration of cfDNA had a worse survival status (log(HR) = 0.60, 95%CI [0.29, 0.90]; **Figure 3C**), with a lower heterogeneity (I^2^ = 59.1%, P = 0.017). Additionally, there is a more significant difference in CRPC,with log(HR) = 0.65, 95%CI [0.33, 0.98] for PFS and log(HR) = 0.59,95%CI [0.34, 0.83] for OS, respectively(**Figures 3B, D**). In order to further explore the relationship of cfDNA with clinical variables in the PCa patients, we collected clinical variables such as Metastasis, Glesson score, and PSA in the current publication. A summary of the overall prognostic value of Metastasis (yes *vs* no, HR = 2.42 (95%CI 0.71, 4.13), n = 5), Glesson score (high *vs* low, HR = 1.17(95%CI 0.96–1.38), n = 5) and PSA (high *vs* low, HR = 1.08(95%CI 1.05–1.12), n = 8) were also presented in **Figure S6**. Subsequently, we focused on the combination of PSA and cfDNA in the Torquato’s cohort. Probably because of the heterogeneity of PSA, **Figures 4A, E** showed that there wasn’t any significant relationship of PSA with PFS and OS. In contrast, cfDNA was significantly related with PFS and OS (**Figures 4B, F**). When combining PSA and cfDNA, the differences of KM curves in high and low combined groups were becoming more obvious in both PFS and OS (**Figures 4C, G**). Time-independent ROC analysis showed that the combination of them were better than single variable (**Figures 4D, H**). As results shown in the meta-analysis, there exists certain heterogeneity in the Alexander et al. (36). and Vandekerkhov et al. (35) cohorts (**Figure S7**).

### Publication Bias

**Figure 5:**
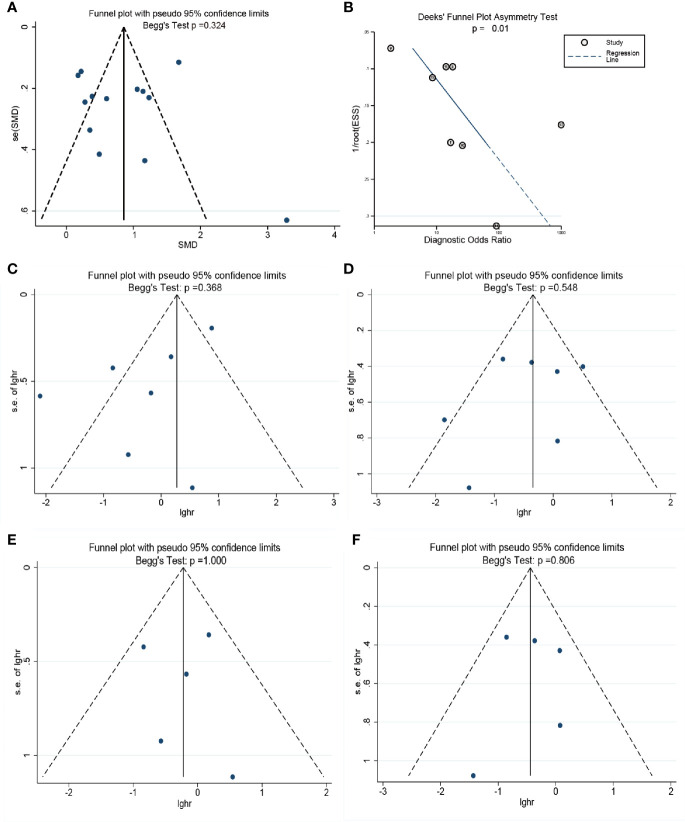
The assessment of potential publication bias in the meta-analysis. **(A)**: The Begg’s test for the pooled results of difference between two groups. **(B)** The Deek’s test for the diagnostic meta-analysis. **(C)**: The Begg’s test for the progression free survival meta-analysis.; **(D)**: The Begg’s test for the overall survival meta-analysis; **(E)**: The Begg’s test for the progression free survival meta-analysis in CRPC; **(F)**: The Begg’s test for the overall survival meta-analysis in CRPC.

The publication bias for each endpoint was accessed with funnel plot. There was no evidence showing the publication bias existed in the pooled difference (P = 0.324) of the concentration of cell-free DNA between cancer groups and control groups. However, Deeks’ funnel plot showed that it may be in the pooled diagnostic value (Deeks P = 0.01). For the pooled PFS and OS, especially in CRPC, the Begg’s test showed there are no publication bias in our meta-analysis with *p* value of 0.368 in the progression free survival meta-analysis (**Figure 5C**), 0.548 in the overall survival meta-analysis (**Figure 5D**), 1.000 in the progression free survival meta-analysis in CRPC (**Figure 5E**), 0.806 in the overall survival meta-analysis in CRPC (**Figure 5F**). The details are shown in **Figure 5**.

### Regression Analysis

Regression analysis in these types of different components in the meta-analysis was performed according to the clinical variables of patients such as Publication year, Age (mean, year), Treatments, Number of patients (n), Early stage (%), Detected Method, Sample source. Results showed that the variables did not influence the meta result (**Table 2**).

**Table 2:** The regression analysis components of the cell-free DNA in the patients with PCA.

Parameter		Publication, year	Age, mean, year	Treatments	Number of patients, n	Early stage,%	Detected Method (QPCR *vs* Others)	Sample source (Serum *vs* Others)
PFS-analysis	B(95%CI)	(−0.9794655–1.503457)	(−0.0871355–0.5698736)	(−1.348412–1.11412)	(−0.0176474–0.0092637)	(−5.154026–8.596189)	(−0.8999634–1.686611)	(−2.408176–4.270278)
	P-value	0.624	0.122	0.824	0.475	0.563	0.485	0.521
OS-analysis	B(95%CI)	(−0.0174465–0.0944092)	(−0.5664279–0.5376342)	(−0.2184801–0.5279275)	(−0.0039293–0.0032868)	/	(−0.3082015–0.3026709)	(−2.249472–0.6187506)
	P-value	0.143	0.951	0.350	0.835	/	0.983	0.214

Early stage,%, mainly according to the AJCC and other systems; PFS, progression-free survival; OS, overall survival.

## Discussion

A growing body of evidence has shown that the serological concentration of cell free-DNA is alleviated in multiple cancers, including lung cancer (38), colon cancer (10, 39) and breast cancer (40, 41) *etc*. The majority of publications that we reviewed focused on the area of detection of tumor specific genomic and epigenetic change in circulating DNA, mostly comprising of CNV (42), mutation and promoter methylation (43, 44). But only in a fraction of cancer patients such genomic changes in circulating DNA can be discovered (19, 45). In contrast, cfDNA level can be measured from a small volume of serum or plasma in all the cancer patients. To our best knowledge, this is the first overall meta-analysis to examine the prognostic value of total cfDNA concentration in patients with PCA.

At present, PSA is considered as the gold standard marker to detect prostate cancer and monitor the tumor progression. Since there is no logical connection between PSA and cfDNA concentration, the result of PSA test might not correlate with cfDNA concentration, as shown in our reexamination of Torquato’s raw data (34). This may add another layer of benefit, since the two tests might compensate each other to overcome the inherent limitations. Our systematic review confirmed that the total cell free-DNA level is significantly higher in PCA patients than in the healthy population as well as in the patients with BPH. In our analysis, from the 14 cohorts included in our study, the quantification of cfDNA has high specificity [AUC = 0.87 (0.84, 0.90)]. Since the PSA test for early prostate cancer screening is lack of specificity and is causing an increase of modality (46, 47), cfDNA concentration might be used together with PSA value to improve the specificity of detection. Torquato’s study (34) has shown the combination of PSA and cfDNA might improve the early detection of PCA.

It is essential in PCA therapy to distinguish patients who have high risk of tumor recurrence. Our study indicated that the high level of cfDNA confers high risk of the disease progression and death rate, particularly in CRPC. Although metastatic castration-resistant prostate cancer (mCRPC) patients generally have an unfavorable prognosis, not all patients have an identical clinical course (48). The patients included in about 50% (11/23) studies of our meta-analysis were undergoing diverse therapies, such as chemotherapy, androgen deprivation therapy, radiotherapy, and prostatectomy (**Table 1**). CfDNA concentrations were tested at the beginning of the therapy. The subgroup and regression analysis indicated that the treatment strategies did not affect the prognostic value of cfDNA in PCA. Since the cfDNA may originate from the micrometastatic sites of tumors, the cfDNA level might be a prognostic determinant for PCA at the time when the treatment was indicated. In addition, from the raw data of 179 patients from three individual cohorts (34–36), we found that incorporation of cfDNA test or the combination with PSA might help to distinguish the patients with recurrent and survivals in Torquato’s cohort study (34) (**Figure 4**).

Even though the biochemical recurrence of PSA has been commonly utilized in the clinics, only the sharp rise of PSA shortly after the treatment is helpful in predicting the treatment failure for PCA patients (49). Our analysis has shown that the cfDNA level has comparable prognosis value, and the combinatorial measurement of total cfDNA concentration and PSA facilitate distinguishing the potential lethal prostate cancers from the indolent ones.

It isn’t surprising that there is significant heterogeneity among these studies. One of the main causes for the heterogeneity is the different methods employed by the studies. The different cfDNA quantification methods, with their pros and cons shown in **Table S8**, might generate incompatible data. Even though the QPCR assay is most commonly employed for cfDNA quantification, this methodology is only adopted in 11 out of the total 23 studies that we assembled in our analysis. It is rather difficult to directly compare the results generated from different methods. Secondly, the concentration of cfDNA is associated with disease progression. This concentration depends on tumor metastatic volume, metastasis sites, and tumor progression. Thus, there is a lack of a well-defined endpoint for the assessment of the clinical interest. In addition, the pre-analytic factors, such as how to process the specimen, also affect cfDNA yield and quality (50). Leukocyte lysis is one of the important factors which can complicate the cfDNA extraction. The blood collecting tubes and centrifuging protocols also greatly affect the cfDNA yield.

Even though there are quite a few cfDNA studies in multiple cancers. Thus far, the only systemic analysis of prognostic value of ctDNA level has been conducted for colorectal cancer (51). In comparison with the analysis in colorectal cancer, the prognostic value of cfDNA in prostate cancer has shown similarly excellent predictive performance. However, the heterogeneity of cfDNA analyses for both cancers are relatively high, which might be due to the same problems existing in the cfDNA tests we discussed above.

In the future, there is an urgent need to implement rigorous clinic trials which utilize the standardized methodology to process the specimen and measure the cfDNA concentration. This might be invaluable to minimize the noise in the ctDNA analyses. The large-scale cohort patient data will help establish the predefined cut-off value for the cfDNA level in PCA patients. Meanwhile, it is essential to specify the clinical endpoints for the cfDNA trials. Arm with the next generation sequencing technology, the cfDNA test is promising to provide biomarkers to select patients more likely benefit from the hormonal and systemic therapy.

## Limitations

The innovation of our research is that we firstly and overall analyzed the diagnostic and prognostic value of all features of cfDNA. However, there are still some limitations in our meta-analysis. Firstly, the pooled results of meta-analysis from different components of cell-free DNA in the articles about how high heterogeneity which cannot be eliminated due to the PCA patients manifesting different clinical features. In addition, many researchers only reported positive results of cell-free DNA, which indicates that publication bias may have influenced our findings for the prognostic values. Furthermore, we cannot fully assess the values of cell-free DNA in current patients with PCA due to lack of liquid biopsy indicators such as mitochondrial DNA and exosomes.

## Conclusions

While this study did not dissolve the heterogeneity, it did partially substantiate the prognostic value of the concentration of cell-free DNA. These findings may help us to understand the significance of cell-free DNA level in the patients with PCA and improve PCA therapeutic strategy in this field.

## Data Availability Statement

All datasets presented in this study are included in the article/**Supplementary Material**.

## Author Contributions

HL and YG contributed equally to this work. All authors contributed to the article and approved the submitted version.

## Conflict of Interest

The authors declare that the research was conducted in the absence of any commercial or financial relationships that could be construed as a potential conflict of interest.
