# Supplementary material for: The Prognostic Value of Plasma Cell-Free DNA Concentration in the Prostate Cancer: A Systematic Review and Meta-Analysis
Source: Front Oncol. 2021 Mar 11;11:599602. doi: 10.3389/fonc.2021.599602 (PMC7991303; doi:10.3389/fonc.2021.599602)
Supplement: Supplementary file 1 [file DataSheet_1.pdf]

Supplementary Materials

These supplementary materials were for the article entitled

“**The Prognostic value of Plasma Cell-free DNA Concentration in the Prostate Cancer: A Systematic Review and Meta-Analysis.**”

Catalogue:

Table S1:Research strategies 3
Table S2: Assessment of study quality 5
Figure S3:The Sensitivity , Specificity and SROC curves for cfDNA in diagnosis of prostate cancer. 7
Figure S4:Diagnosis of total concentration in the Pooled DOR,NLR and PLR for PCA. 8
Figure S5:The pooled results of biochemical recurrence free survival in the patients with PCA 9
Figure S6: The comparison of other variables with the concentration of cfDNA in PCa 10
Figure S7: The prognosis value of the combination of PSA and cfDNA for the patients with PCa. 11
Table S8: The pros and cons of different cfDNA quantification methods. 13

Figure Legends

Table S1: Research strategies for meta-analysis

Table S2: Assessment of study quality

Figure S3: The Sensitivity(S3A) , Specificity(S3B) and SROC curves(S3C) for cfDNA in diagnosis of prostate cancer.

Figure S4: Diagnosis of total concentration in the Pooled DOR,NLR and PLR for PCA.

Figure S5: The pooled results of biochemical recurrence free survival in the patients with PCA.

Figure S6: The comparison of other variables with the concentration of cfDNA in PCA

Figure S7: The prognosis value of the combination of PSA and cfDNA for the patients with PCA in Wyatt's and Vandekerkhov's article；S7A: PSA for PFS in Wyatt's article; S7B: CfDNA for PFS in Wyatt's article;S7C: PSA+cfDNA for PFS in Wyatt's article; S7D: PSA for OS in Vandekerkhov's article; S7E: CfDNA for OS in Vandekerkhov's article; S7F: PSA+cfDNA for OS in Vandekerkhov's article; S7G: Time-dependent ROC of them for predicting PFS in Wyatt's article; S7H: Time-dependent ROC of them for predicting OS in Vandekerkhov's article.

Table S8: The pros and cons of different cfDNA quantification methods.

Table S1:Research strategies

Databases	Total citations	Full text
**PubMed**	**911**	**124**
**Cochrane Library**	**12**	**3**
**Web of science**	**56**	**8**
**Embase**	**58**	**5**
**PMC**	**9**	**1**
**Otherdatabase source**	**63**	**6**
**Total(database)**	**1109**	**147**

For the meta-analysis, the authors searched PubMed, Web of Science, Medline, PMC,EMBASE and the Cochrane Library to retrieve all eligible articles from the date of database inception to June 30, 2020. The search heading terms and keywords included “prostate cancer”; “cf DNA” ,“diagnosis” and “prognosis”. Additional articles were identified by manually reviewing the references of included articles. No languages restrictions were applied. Others details could see the following .

Explicit search strategy:

steps	Strategy
**Diagnosis OR prognosis**	Search **((((((((((((Diagnoses) OR (Diagnoses and Examinations)) OR (Examinations and Diagnoses)) OR correlation) OR relation) OR correlationship) OR prognosis) OR diagnosis) OR Prognostic Factors) OR Factor, Prognostic) OR Factors, Prognostic) OR Prognostic Factor) OR prognoses**
**cfDNA**	Search **(((((((((((((((((((((((((((((((Cell Free Nucleic Acids) OR Nucleic Acids, Cell-Free) OR Circulating Cell-Free Nucleic Acids) OR Circulating Cell Free Nucleic Acids) OR Circulating Nucleic Acids) OR Acids, Circulating Nucleic) OR Nucleic Acids, Circulating) OR Cell-Free Nucleic Acid) OR Cell Free Nucleic Acid) OR Nucleic Acid, Cell-Free) OR Cell-Free DNA) OR Cell Free DNA) OR DNA, Cell-Free) OR cfDNA) OR cirDNA) OR Cell-Free Deoxyribonucleic Acid) OR Acid, Cell-Free Deoxyribonucleic) OR Cell Free Deoxyribonucleic Acid) OR Deoxyribonucleic Acid, Cell-Free) OR Circulating DNA) OR DNA, Circulating) OR Cell-Free RNA) OR Cell Free RNA) OR RNA, Cell-Free) OR cfRNA) OR cirRNA) OR Cell-Free Ribonucleic Acid) OR Acid, Cell-Free Ribonucleic) OR Cell Free Ribonucleic Acid) OR Ribonucleic Acid, Cell-Free) OR Circulating RNA) OR RNA, Circulating**

**prostate cancer**	Search **((((((((((((((((Prostate Neoplasms) OR Neoplasms, Prostate) OR Neoplasm, Prostate) OR Prostate Neoplasm) OR Neoplasms, Prostatic) OR Neoplasm, Prostatic) OR Prostatic Neoplasm) OR Prostate Cancer) OR Cancer, Prostate) OR Cancers, Prostate) OR Prostate Cancers) OR Cancer of the Prostate) OR Prostatic Cancer) OR Cancer, Prostatic) OR Cancers, Prostatic) OR Prostatic Cancers) OR Cancer of Prostate**
**Ultimate**	total

Table S2: Assessment of study quality

Detailed Newcastle-Ottawa Scale of each included case- control study

ID		Selection				Comparability		Exposure			
Cations	References	Case definition	Representative of case	Control selection	Control definition	Main factor	Associated factor	Exposure ascertainment	Same method of ascertainment	Non-response rate	Total score
(18)	Boddy,et al (2005)	*	*	*	*	*		*	*		7
(20)	Altimari,et al (2008)	*	*	*	*	*		*	*		7
(22)	Cherepanova,et al (2008)	*	*	*	*	*			*		6
(25)	Goodall,et al (2017)	*	*	*		*	*	*		*	7
(14)	Hendriks.et al (2018)	*	*	*	*	*	*	*	*	*	9
(27)	Reis,et al (2015)	*	*	*		*		*	*	*	7
(28)	Jung.et al (2004)	*	*	*	*	*	*	*	*		8
(30)	Wroclawski.et al (2013)	*	*	*	*	*	*	*	*		8
(31)	Mehra.et al (2006)	*	*	*	*	*			*		6
(32)	Papadopoulou,et al (2006)	*	*	*	*	*			*		6

Detailed Newcastle-Ottawa Scale of each included cohort study

ID		Selection				Comparability		Outcome			
**Cations**	**References**	**representativesof exposed cohort**	**Selection of non-exposed cohort**	**Ascertainment of exposure**	**Demonstration that outcome of interest was not present at start of study**	**Adjust for the most important risk factors**	**Adjust for other risk factors**	**Assessment of outcome**	**Follow-up length> 10 years**	**Adequacy of follow-up rate**	**Total score**
**(19)**	**Allen , et al (2004)**	*	*	*	*	*		*			**6**
**(21)**	**Belic,et al (2018)**	*	*	*	*	*	*	*			**7**
**(23)**	**Chun,et al (2006)**	*	*	*	*	*	*	*			**7**
**(24)**	**Ellinger.et al (2008)**	*	*	*	*	*	*	*			**7**
**(26)**	**Gordian,et al (2010)**	*	*	*	*	*	*	*			**7**
**(29)**	**Kienel,et al (2015)**	*	*	*	*	*	*	*			**7**
**(33)**	**Ponti.et al (2018)**	*	*	*	*	*	*	*			**7**
**(34)**	**Schutz,et al (2015)**	*	*	*	*	*	*	*			**7**
**(35)**	**Schwarzenbach,et al (2009)**	*		*	*	*	*	*			**6**
**(36)**	**Torquato, et al (2019)**	*	*	*	*	*	*	*			**7**
**(17)**	**Vandekerkhov, et al (2018)**	*	*	*	*	*		*			**6**
**(16)**	**Wyatt,et al (2016)**	*	*	*	*	*	*	*			**7**
**(37)**	**Annala,et al (2018)**	*	*	*	*	*	*	*			**7**

Figure S3:The Sensitivity , Specificity and SROC curves for cfDNA in diagnosis of prostate cancer.

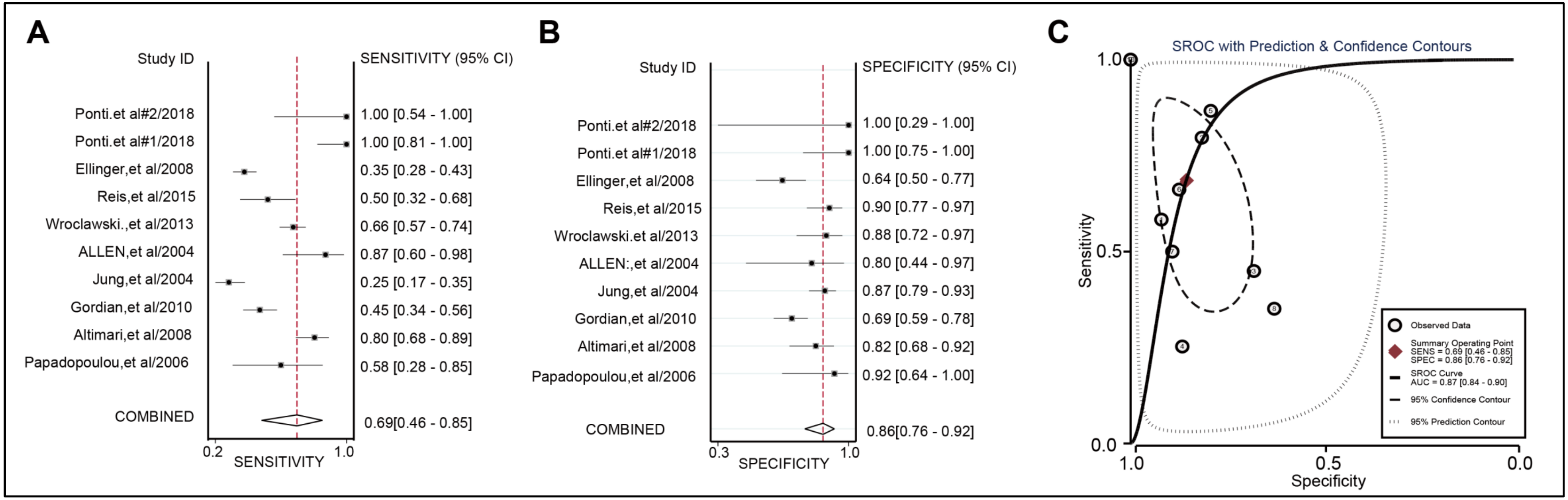

Figure S4:Diagnosis of total concentration in the Pooled DOR,NLR and PLR for PCA.

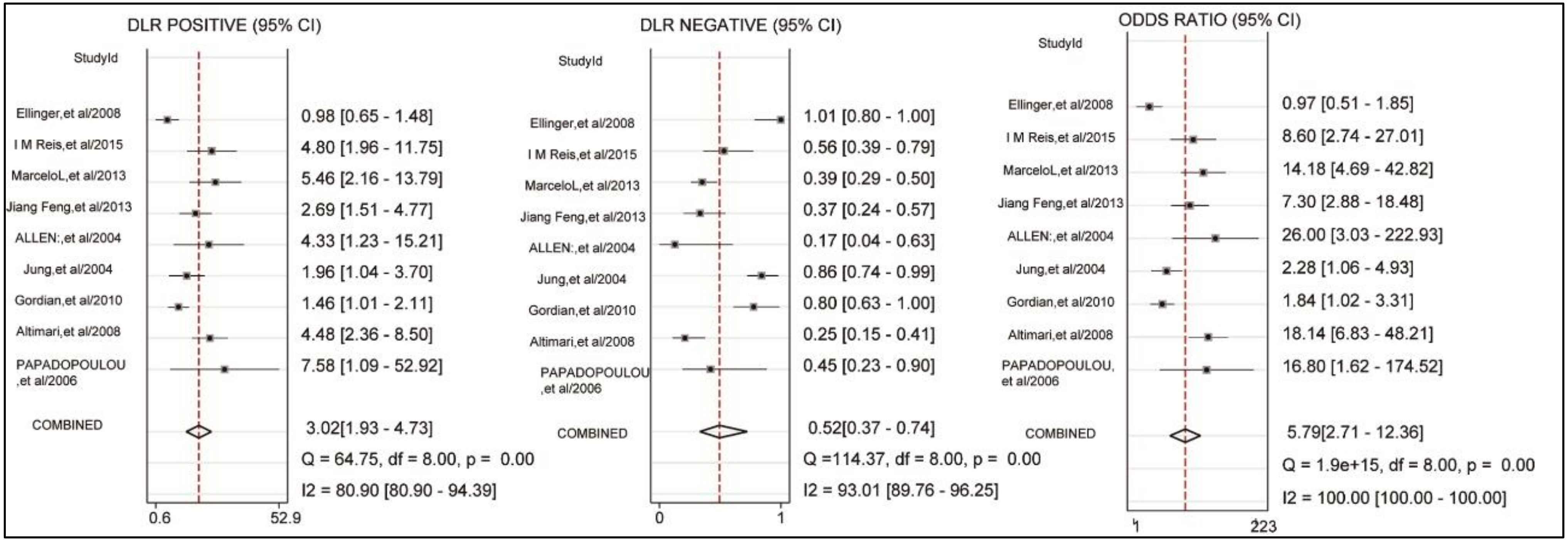

Figure S5:The pooled results of biochemical recurrence free survival in the patients with PCA

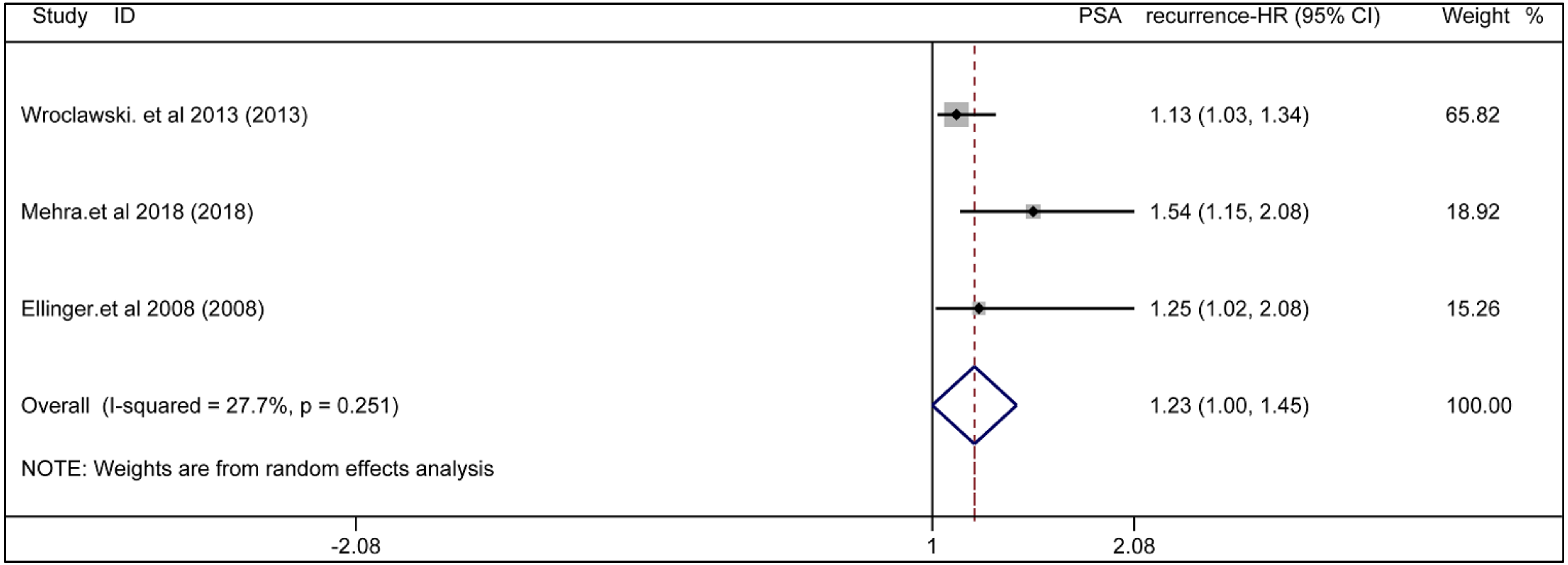

Figure S6: The comparison of other variables with the concentration of cfDNA in PCa

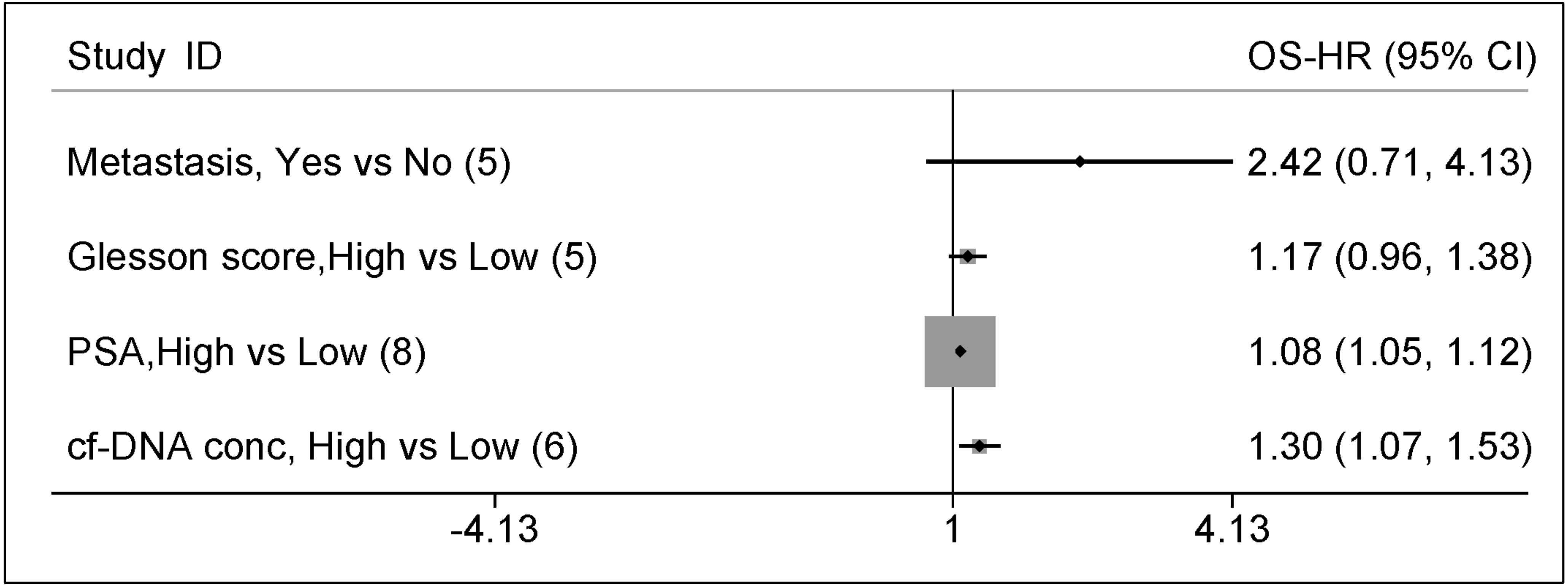

Figure S7: The prognosis value of the combination of PSA and cfDNA for the patients with PCa.

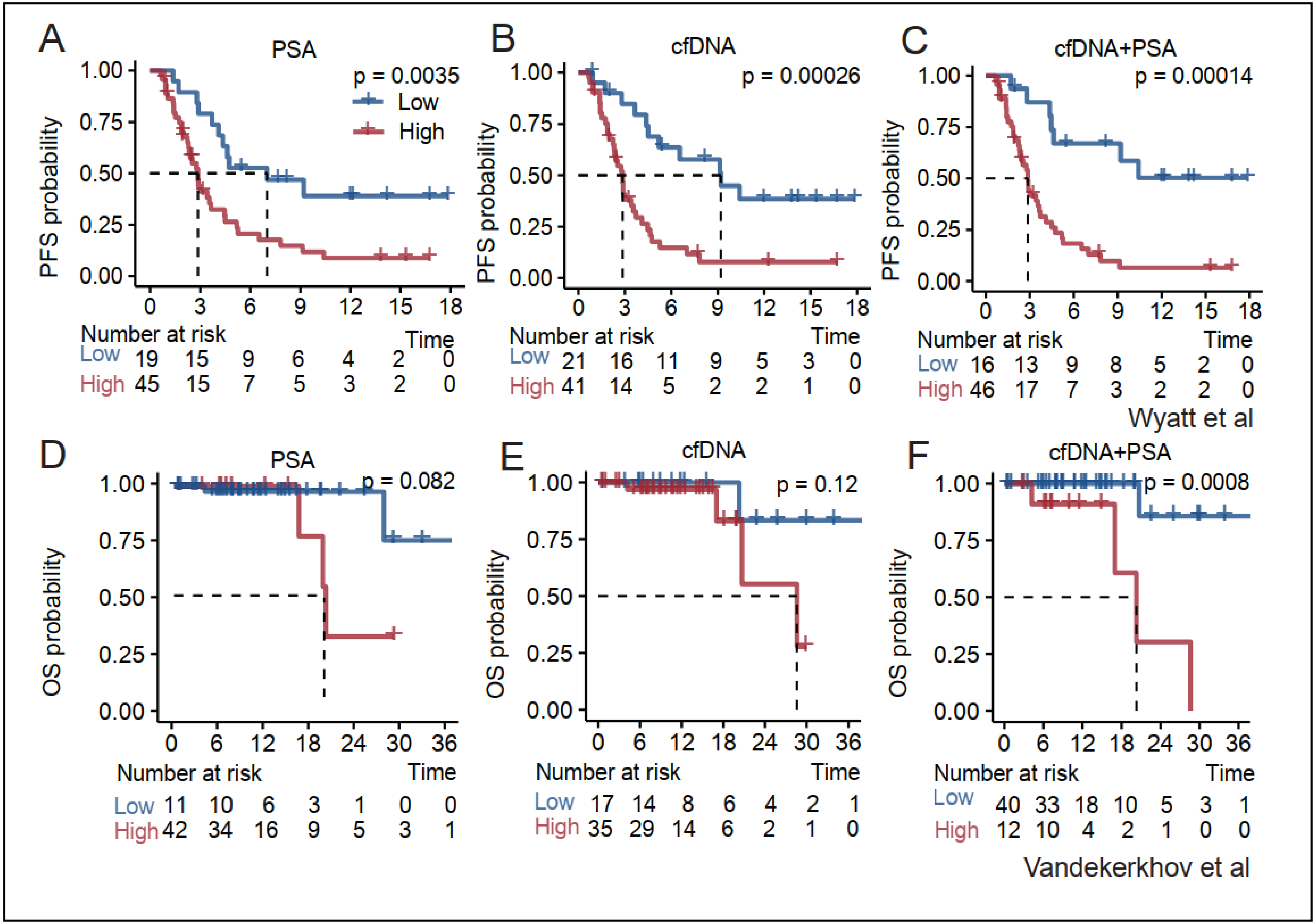

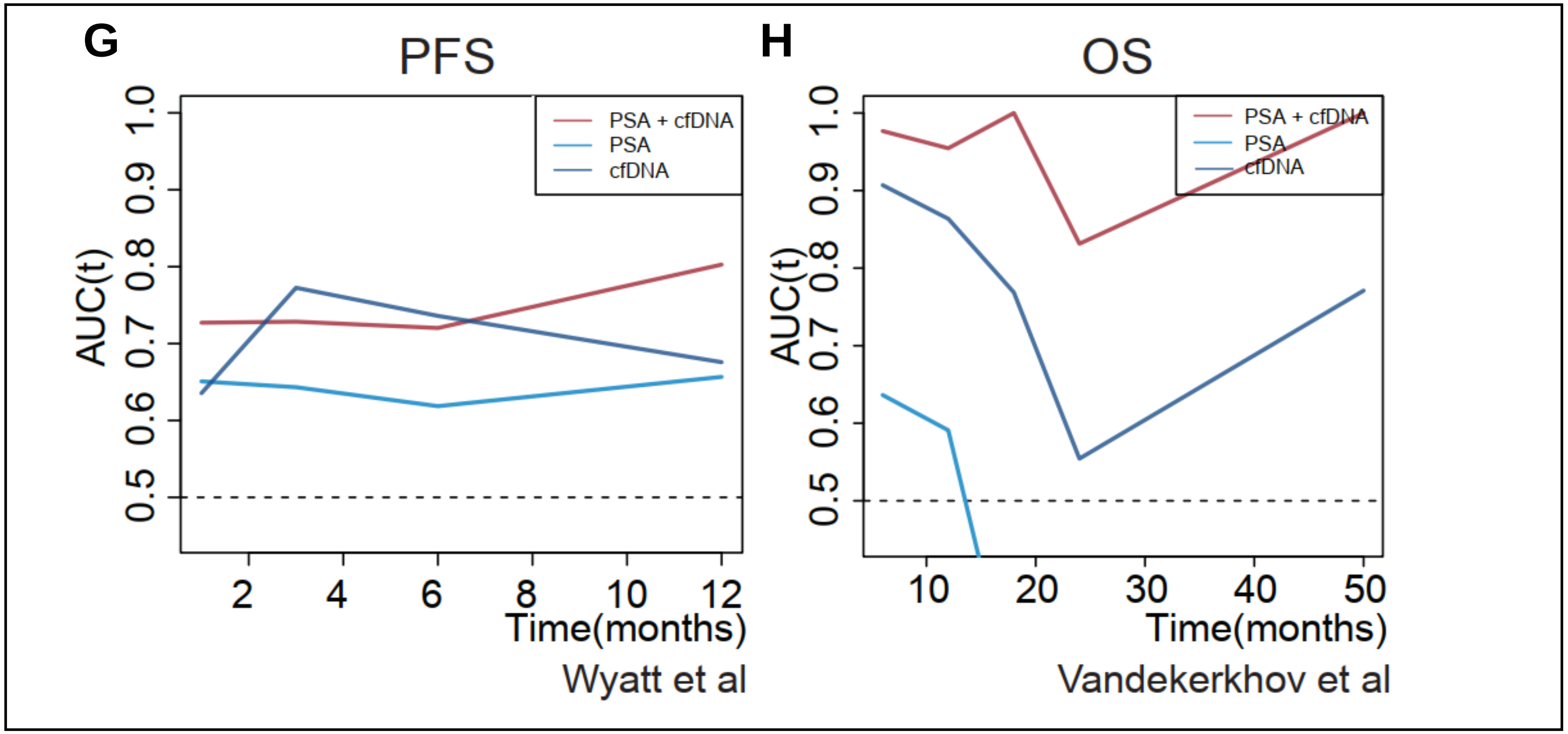
G
PFS
H
OS
PSA + cfDNA
PSA
cfDNA
AUC(t)
0.5
0.6
0.7
0.8
0.9
1.0
2
4
6
8
10
12
Time(months)
Wyatt et al
10
20
30
40
50
Time(months)
Vandekerkhov et al

Table S8: The pros and cons of different cfDNA quantification methods.

Methods		Pros	Cons	citations
Spectrophotometric methods:NanoDrop		Inexpensive;<br>Easy to perform;<br>Be used to estimate the quality of a sample	Cannot distinguish between dsDNA, ssDNA, RNA, oligonucleotides, and free nucleotides;<br>Have a poor predictive ability	[23],[29],[35]
Fluorometric methods	SYBRGreen I or PicoGreen	More accurate and more sensitive compared to spectrophotometry technics	More expensive;<br>Lower accuracy	[22],[25],[30],
	Qubit	Fast, cost-effective	Lower accuracy	[14],[33]
Quantitative real-time PCR.	QPCR,digital PCR	Fast,high technical sensitivity, high precision	Expensive,The accuracy can be affected by fragmentation,	[18],[19],[20],[21],[24],[26],[27], [28], [31],[32]
Next Generation Sequencing		More sensitive; More precise	More expensive	[16],[17],[34],[36],[37]
